# A rare case report of reversible glucose counterregulation in an insulinoma patient with type 2 diabetes

**DOI:** 10.1007/s12020-024-03703-9

**Published:** 2024-02-09

**Authors:** Jian-hui Teng, Jun-pei Hu, Xia Wang, Chi Zhang, Jing Chen

**Affiliations:** 1 Hunan Provincial People’s Hospital, The First Affiliated Hospital of Hunan Normal University, Changsha, 41005 Hunan Province China; 2 Department of Endocrinology, Hunan Provincial People’s Hospital, The First Affiliated Hospital of Hunan Normal University, Changsha, 41005 Hunan Province China

**Keywords:** Insulinoma, Type 2 diabetes (T_2_DM), Adrenocortical insufficiency, Glucose counterregulation, Case report

## Abstract

**Context:**

Insulinoma is a neuroendocrine tumor derived from pancreatic β -cells whose clinical manifestation is recurrent hypoglycemia. Insulinoma in a patient with preexisting diabetes is extraordinarily rare, and the unmasking of type 2 diabetes (T_2_DM) after insulinoma surgery is even rarer.

**Case report:**

This article reports a 49-year-old male patient with insulinoma that masked the diagnosis of T_2_DM. The patient was admitted to the hospital with symptoms of hypoglycemia, such as repeated sweating, palpitations, and asthenia for over 4 years. The patient was diagnosed with insulinoma after completing relevant examinations. The emergence of hyperglycemia after the removal of insulinoma is attributable to the coexistence of T_2_DM. Surprisingly, a reversible decrease in cortisol levels was observed during the diagnostic process. We searched the previously published reports of this type of case from PubMed to determine why type 2 diabetes was covered by insulinoma and why glucocorticoids decreased.

**Conclusions:**

The diagnosis of T_2_DM in the patient after surgery may be related to increased food intake and insulin resistance induced by hyperinsulinemia caused by long-term hypoglycemia. The reversible decrease in cortisol levels, not adrenocortical insufficiency during the diagnostic process, may be caused by a transient abnormality in glucose counterregulation.

## Introduction

Insulinoma is the most common pancreatic neuroendocrine tumor and the most common cause of endogenous hyperinsulinemia hypoglycemia. However, its incidence rate is extremely low, approximately 1–4/1 million people annually [1–3]. Although up to 16% of insulinomas are malignant, they are usually benign, small, and isolated tumors, with a few being type 1 multiple endocrine tumors (MEN-1). MEN1 syndrome has genetic susceptibility and is associated with loss of 11q13 heterozygosity [4], which is characterized by the association of primary hyperparathyroidism, pituitary adenomas and gastric/pancreatic tumors such as gastrinomas or insulinomas [5].

Insulinoma is a disease characterized by excessive insulin secretion causing hypoglycemic symptoms, including autonomic nervous system excitatory symptoms and central nervous system dysfunction. It mainly manifests as the typical Whipple’s triad, including hypoglycemia, neuroglycopenic symptoms, and relief of symptoms following glucose intake. The crucial biochemical characteristic of insulinoma is endogenous hyperinsulinemic hypoglycemia (EHH). Hypoglycemic symptoms of insulinoma often occur on an empty stomach, and patients with atypical symptoms must complete a starvation test. Insulinoma can be excluded if the 72-h starvation test fails to induce hypoglycemia.

The incidence of insulinoma is extremely low, and the chances of two endocrine diseases with contradictory effects, insulinoma and diabetes, occurring in the same patient are even rarer. Under normal physiological conditions, the body’s blood glucose balance is maintained by the balanced regulation of glucose-increasing and glucose-lowering hormones. When hypoglycemia occurs, the hypothalamic-pituitary-adrenal axis (HPA axis), the body’s blood glucose counterregulatory mechanism, is activated, resulting in increased secretion of glucose-raising hormones, including glucagon, catecholamines, cortisol, and growth hormone, which raise blood glucose by regulating hepatic glycogenolysis and peripheral blood glucose utilization. Long-term hypoglycemia can lead to abnormal glucose counterregulation and secondary adrenal insufficiency. These patients need to be supplemented with glucocorticoids before surgery; otherwise, they will have life-threatening adrenal crisis, and their cortisol function will return to normal after surgery. We report a rare case of T_2_DM in a patient with insulinoma who had a reversible preoperative decrease in cortisol levels and transient hypothalamic-pituitary-adrenal axis dysfunction. Through a literature review of previous cases, we summarized the clinical characteristics and key points of diagnosis and treatment for insulinoma.

## Case report

A 49-year-old male patient had recurrent sweating, palpitations and asthenia episodes for nearly 5 years, which were completely relieved after eating. In the first four years, these symptoms occurred about 1–3 times a year and are relatively mild. Due to a lack of health awareness and the patient’s complete relief after eating, the patient only paid attention to these symptoms once they became more and more frequent and severe in the past year. At this point, the patient began seeking help from local clinics but was limited to testing fingertip blood sugar. The patient’s fingertip blood glucose fluctuates between 3 and 5 mmol/L each time the above symptoms occur. Every time symptoms occur, the patient alleviates them through eating, resulting in a weight gain of 25 kg. At 6 am five days earlier, the patient had suddenly altered consciousness and could not recognize people. At that time, he was immediately sent to the local hospital, in addition to measuring a fasting venous blood glucose level of 1.7 mmol/L, it was also found that his fasting insulin and fasting C-peptide were elevated. The hospital considered the possibility of diagnosis as insulinoma, but abdominal CT scan did not detect the tumor. In order to seek further diagnosis and treatment, he went to our hospital for treatment. The patient had no history of diabetes mellitus, other medical conditions or long-term medication. None of his parents, grandparents, and siblings had diabetes or endocrine tumors. Vital signs on presentation indicated a body temperature of 36.7 °C, heart rate of 69 beats/min, blood pressure of 130/90 mmHg, and respiratory rate of 20/min. His current weight, height, and body mass index (BMI) were 172 cm, 115 kg, and 38.87 kg/cm^2^, respectively. Acanthosis nigricans signs were seen on the neck and axilla. This patient had extremely dark areolas and hyperpigmented skin on his hands and lower limbs.

**Fig. 1:**
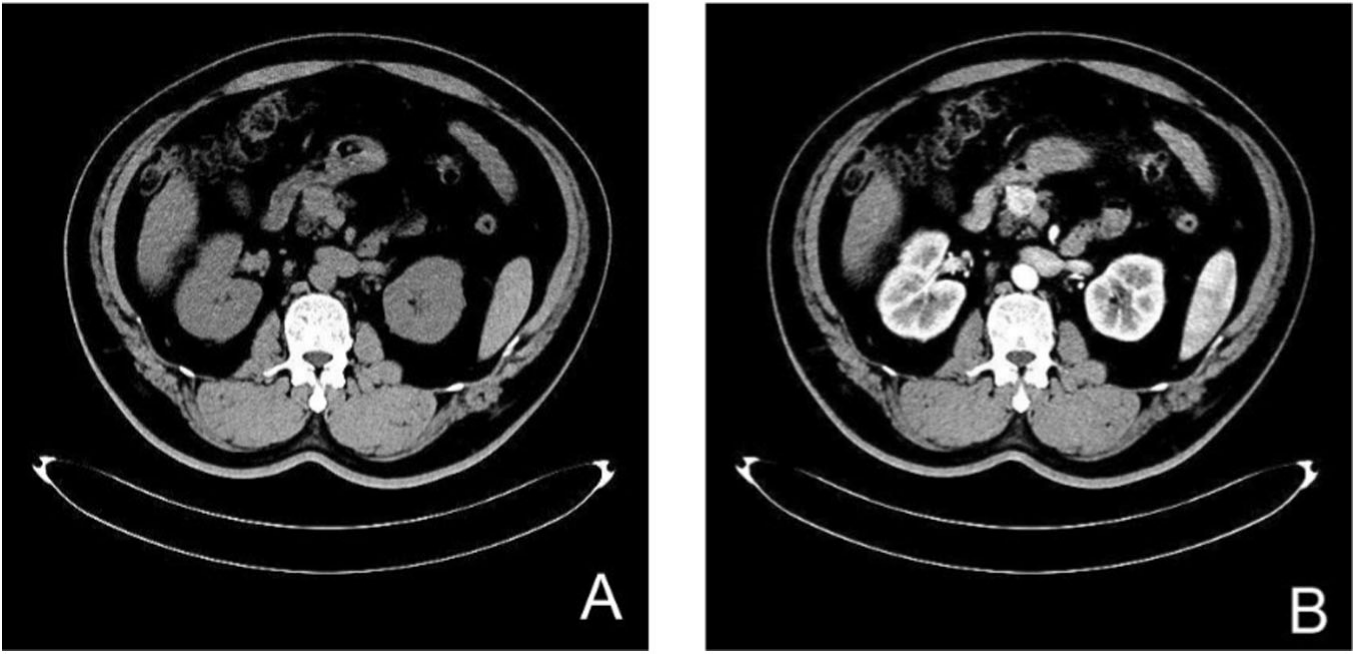
Imaging examination of insulinoma. **A** CT indicated a 2 × 3 cm occupancy at the junction of the pancreatic head and neck; (**B**) the occupancy with vascularity in CT enhanced scan

**Fig. 2:**
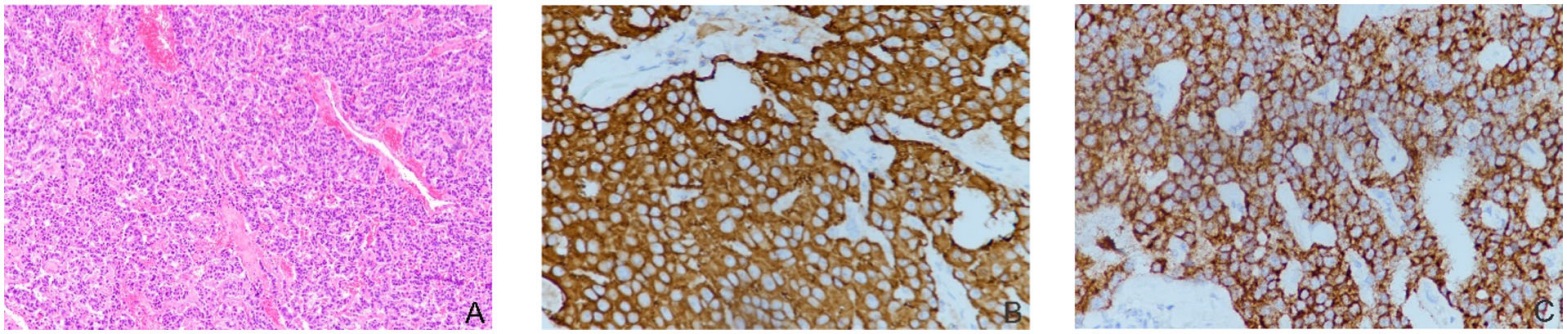
Histopathological analysis and immunohistochemical examination of the resected specimen. **A** Tumor cells arrange in solid cellular sheet and form a pseudoglandular structure (haematoxylin and eosin staining; magnification ×100); (**B**) positive immunohistochemical staining for chromogranin A (×400); (**C**) positive immunohistochemical staining for synaptophysin (×400)

The patient’s laboratory examination indicated that tumor markers were standard, and parathyroid hormone, blood calcium level, six sex hormones, adrenocorticotropic hormone level, IGF-1, and IGFBP-3 were within the normal range. Serum insulin and C-peptide were significantly increased during hypoglycemia (Table 1), along with a shallow cortisol level (Table 2). The patients’ cortisol levels reached 1.75 μg/dl after glucose supplementation to maintain blood glucose. Computed tomography (CT) scans indicated a 2*3 cm occupancy with abundant blood supply at the junction of the pancreatic head and neck (Fig. 1), and the diagnosis of insulinoma was considered. The patient underwent laparoscopic pancreatic mass dissection, during which a 3*2.5*2 cm-sized mass in the middle part of the pancreas was dissected. The immunohistochemical evaluation showed positivity for chromogranin A, synaptophysin, CD56, and CKP (Fig. 2), and a ki67 proliferation index of 3%. Postoperatively, the patient’s cortisol returned to normal at 8:00 am, at 19.69 μg/dl (normal value: 4.2–24.8 μg/dl) and 38.73 pg/ml (normal value: 7.2–63.4 pg/ml) of adrenocorticotropic hormone. In addition, the sudden rise in blood glucose in postoperative patients lasts 6 days, and even blood glucose can reach more than 20.0 mmol/L. The patient had normal glycosylated hemoglobin and was negative for immune antibodies for diabetes. Combined with the clinical immunophenotype and clinical data, this patient was diagnosed with insulinoma and T_2_DM. Postoperatively, the patient had no symptoms of pancreatic exocrine insufficiency and was treated with insulin pump for one week. One week after surgery, the patient began oral administration of pioglitazone and metformin (15 mg/500 mg, twice a day), as well as subcutaneous injection of Insulin Degludec (18iu) before bedtime for hypoglycemic treatment.

**Table 1:** Patient’s blood test results

Test	Level	Criterion for EHH	Comment
Laboratory glucose, mmol/L	0.95	≤3.0	Low
Insulin, mU/L	230.30	≥3.0	Elevated
C-peptide, nmol/L	49.29	≥0.2	Elevated

*EHH* endogenous hyperinsulinaemic hypoglycaemia

**Table 2:** Patient’s blood test results

Test	Level	Reference interval	Comment
Cortisol (08:00)	0.99	4.2–24.8	Low
Cortisol (16:00)	0.32	2.9–17.3	Low
Cortisol (24:00)	0.29	0–6.7	Low
ACTH (08:00)	19.04	7.2–63.4	Normal range
ACTH (16:00)	5.50	3.0–32.0	Normal range
ACTH (24:00)	4.84	0–32.0	Normal range

After discharge, we followed up with the patient. After 3 months, the measured values of fasting and postprandial blood glucose levels in patients were 6.2 mmol/L, 9.4 mmol/L. She had lost almost 12 kg during the 3month and reduced the bedtime insulin to 8 iu. Considering that the patient did not follow the diabetes diet and exercise properly, local physicians decided to wait for 3 months without changing the treatment plan. When the patient visited again 3 months later, his BMI was 31 kg/m^2^ and insulin had been discontinued, only oral medication was used for treatment. At this time, the patient’s fasting and postprandial insulin levels were 5.0 mmol/L, 7.0 mmol/L. One week ago, nine months after surgery, we followed up with the patient again, she has lost a total of 25 kg of weight and her fasting and postprandial blood glucose levels at that time were 5.4 mmol/L, 7.8 mmol/L. Currently, he is receiving treatment with pioglitazone and metformin, and his blood sugar is well controlled without experiencing hypoglycemia. The investigations and treatment profiles of the patient are summarized in Table 3.

**Table 3:** The follow-up profiles of the patient

	Pre-operative	Post-Treatment		
		3 months	6 months	9 months
Insulin, mU/L	230.30	ND	ND	ND
C-peptide, nmol/L	49.29	ND	ND	ND
Fasting plasma glucose, mmol/L	0.95	6.2	5.0	5.4
Postprandial plasma glucose, mmol/L	–	9.4	7.0	7.8
BMI (kg/m^2^)	38.87	34.82	31.77	30.42
Treatment	–	pioglitazone and metformin (15 mg/500 mg, twice a day), degludec insulin (8iu)	pioglitazone and metformin (15 mg/500 mg, twice a day)	pioglitazone and metformin (15 mg/500 mg, twice a day)

*ND* not determined

## Discussion

Insulinoma has an insidious onset characterized by isolated solitary, small tumor size, and high benign rate. Laboratory tests at the time of the attack are characterized by endogenous hyperinsulinemia, and clinical manifestations are divided into two categories: one is autonomic nervous system excitatory symptoms caused by increased catecholamines, such as palpitations, hand tremors, pallor, and sweating, and the other is central nervous system dysfunction caused by lack of glucose in brain tissue, such as personality and behavioral changes, coma and epilepsy. The correct diagnosis of insulinoma is crucial because the clinical symptoms of insulinoma are complex and variable and can be easily misdiagnosed. The current qualitative diagnosis of insulinoma relies on Whipple’s triad and EHH. Meanwhile, Whipple’s triad is not unique to insulinoma but also exists in cases of hypopituitarism, adrenocortical insufficiency, severe liver disease or kidney disease, and the use of drugs with hypoglycemic side effects. This patient, in this case, mostly has hypoglycemic episodes in the early morning when fasting, and they will have recurrent hypoglycemia soon after meals without continuing to eat, so there is no need to perform the starvation test. On the one hand, the patient had the classic Whipple’s triad, allowing for a definitive hypoglycemia diagnosis. On the other hand, the significant elevation of serum insulin and C-peptide during hypoglycemic episodes indicates the presence of EHH. Based on comprehensive analysis, the diagnosis of insulinoma can be made qualitatively. Since surgical resection is the only cure for insulinoma, a qualitative diagnosis requires further localized diagnosis.

Insulinoma is rare in patients with prior diabetes and even rarer in patients with diabetes found after insulinoma surgery, making it easy to miss and misdiagnose [6]. The association between insulinoma and diabetes mellitus is unclear. However, some articles suggest a strong association between insulinoma and familial diabetes, and mutations in the MAFA gene may induce diabetes or insulinoma [7]. In addition, 30% of insulinoma patients have mutations in the YY1 gene [8], which regulates the transcription of CXCL12 with antidiabetic potential [9]. Animal experiments have found that overdosing of glargine insulin for 8 weeks in mice with normal glucose tolerance also induced insulin resistance and type 2 diabetes [10].

In a Mayo Clinic study that reviewed 313 patients with insulinoma between 1927 and 1992, only one patient had concurrent diabetes [11]. Cases of diabetes mellitus with insulinoma have also been reported less frequently in the national and international literature [12–15]. The mechanism may be related to increased eating and insulin resistance due to long-term chronic hyperinsulinemia caused by insulinoma [7]. The patient we reported had gained 25 kg over five years, had a BMI greater than 28 kg/cm2, and had acanthosis nigricans in the neck and axilla, suggesting possible insulin resistance. On the one hand, chronic insulinoma secretes too much insulin, causing patients to experience recurrent hypoglycemia, while recurrent episodes of hypoglycemia cause patients to avoid hypoglycemic symptoms by increasing their food intake. Insulin inhibits lipolysis and promotes fatty acid synthesis resulting in ectopic deposition of fat in nonfat organs, releasing excess ROS and proinflammatory factors, which cause systemic inflammation. Prolonged low-grade systemic inflammation prevents insulin from acting in the insulin signaling pathway, disrupting glucose homeostasis and leading to systemic dysregulation [8]. On the other hand, the downregulation of insulin receptor phosphorylation levels in the case of long-term hyperinsulinemia decreases insulin sensitivity [9, 10], which may also contribute to the insulin resistance caused by long-term chronic hyperinsulinemia. Therefore, we conclude that patients experience insulin resistance due to high calorie intake stimulated by hypoglycemia, which is masked by insulinomas and then becomes apparent after tumor resection. We can speculate that due to insulin resistance, the symptoms of hypoglycemia are not severe for a long time, which is why there is a long time from diagnosis to initial symptoms. It can be suggested that the weight loss of 25 kg after surgery will lead to the improvement of insulin resistance and blood sugar level, and lead to the change of treatment strategy for diabetes.

Hypoglycemia-induced excitatory symptoms of the autonomic nervous system are a robust stressor leading to rapid HPA axis activation. Typically, when the plasma glucose concentration decreases, first, pancreatic beta cells stop insulin, followed by increased secretion of glucagon and adrenaline to prevent hypoglycemia. In the case of prolonged hypoglycemia, the secretion of cortisol and growth hormone also increases [16]. Reviewing the literature, we found that patients with insulinoma have reduced cortisol and adrenocorticotropic hormone levels due to secondary adrenocortical insufficiency that can result from chronic hypoglycemia. Moreover, these patients usually require glucocorticoid supplementation before insulinoma surgery to maintain blood glucose and avoid adrenal crisis. Additionally, as postoperative hypoglycemia disappears and glucocorticoid supplementation is gradually reduced, cortisol and adrenocorticotropic hormone levels can be restored, as confirmed in the literature [17]. This may be because the patient’s chronic hypoglycemia has damaged the neurons in the brain that perceive the lowering of blood glucose, making the patient’s hypoglycemic symptoms less obvious, and the feedback regulatory system is not activated sensitively, affecting the function of the HPA axis [18]. In contrast, the role of the HPA axis in glucose regulation was still present in this patient, and the diagnosis of hypoadrenocorticism had not yet been reached. In our case, it was found that the patient’s cortisol level was extremely low, and the adrenocorticotropic hormone level was in the normal range (Table 2). Combined with the patient’s dark areola color and hyperpigmentation of the skin of both hands and lower extremities, we suspected a combination of adrenocortical insufficiency. After the patient was diagnosed with insulinoma, we administered intravenous glucose supplementation 24 h a day and successfully prevented the patient from having an episode of hypoglycemia. Surprisingly, however, we suddenly found that the cortisol levels of the patients retested after glucose supplementation to maintain blood glucose, although not back to normal, were significantly higher than before, indicating that the HPA glucose regulation axis was working. Overall, we concluded that the patient did not have secondary adrenocortical insufficiency but rather a transient dysregulation of the HPA axis. This suggests that a single decrease in cortisol levels during fasting hypoglycemia is less specific for the diagnosis of adrenocortical insufficiency, and further refinement of the ACTH excitation test is needed to clarify the diagnosis.

## Conclusion

In summary, the incidence of insulinoma is low, the clinical manifestations are complex and varied, and some patients have atypical symptoms, which are easily misdiagnosed and mistreated. Therefore, it is crucial to diagnose insulinoma correctly. In addition, monitoring changes in blood glucose after insulinoma surgery to avoid the development of missed postoperative diabetes is important. Last but not least, patients with chronic hypoglycemia should be screened for adrenocortical function in a timely manner to help control hypoglycemia and the safe performance of insulinoma surgery.

## References

[1] Davi MV (2017). The treatment of hyperinsulinemic hypoglycaemia in adults: an update. J. Endocrinol. Invest.

[2] Service FJ (1995). Hypoglycemic disorders. N. Engl. J. Med.

[3] Service FJ (1991). Functioning insulinoma–incidence, recurrence, and long-term survival of patients: a 60-year study. Mayo Clin. Proc..

[4] Oberg K, Skogseid B, Eriksson B (1989). Multiple endocrine neoplasia type 1 (MEN-1). Clinical, biochemical and genetical investigations. Acta Oncol..

[5] Brandi ML (2021). Multiple endocrine neoplasia type 1: latest insights. Endocr. Rev..

[6] Kumar S, Melek M, Rohl P (2022). Case report: hypoglycemia due to metastatic insulinoma in insulin-dependent type 2 diabetes successfully treated with 177 Lu-DOTATATE. Front Endocrinol. (Lausanne).

[7] Shanik MH (2008). Insulin resistance and hyperinsulinemia: is hyperinsulinemia the cart or the horse?. Diabetes Care.

[8] Ahmed B, Sultana R, Greene MW (2021). Adipose tissue and insulin resistance in obese. Biomed. Pharmacother..

[9] Youngren JF (2007). Regulation of insulin receptor function. Cell Mol. Life Sci..

[10] Catalano KJ (2014). Insulin resistance induced by hyperinsulinemia coincides with a persistent alteration at the insulin receptor tyrosine kinase domain. PLoS One.

[11] Kane LA (1993). *I*nsulinoma in a patient with NIDDM. Diabetes Care.

[12] Ademoğlu E (2012). Type 2 diabetes mellitus in a patient with malignant insulinoma manifesting following surgery. Diabet. Med.

[13] Oikawa Y (2012). Insulinoma may mask the existence of Type 1 diabetes. Diabet. Med.

[14] Assyov Y (2016). Concomitant insulinoma and type 2 diabetes mellitus diagnoses: a case report. J. Diabetes.

[15] Ng HY, Pape A (2018). Insulinoma: important in the differential diagnosis of persistent hypoglycaemia unrelated to diabetes. Med J. Aust..

[16] Reno CM (2013). Defective counterregulation and hypoglycemia unawareness in diabetes: mechanisms and emerging treatments. Endocrinol. Metab. Clin. North Am..

[17] Tang HY, Garcia JM (2010). Association between insulinoma and adrenal insufficiency: a case report and review of the literature. Pancreas.

[18] Song Z, Routh VH (2006). Recurrent hypoglycemia reduces the glucose sensitivity of glucose-inhibited neurons in the ventromedial hypothalamus nucleus. Am. J. Physiol. Regul. Integr. Comp. Physiol..

